# Prenatal Relocation Stress Enhances Resilience Under Challenge in Infant Rhesus Macaques

**DOI:** 10.3389/fnbeh.2021.641795

**Published:** 2021-03-29

**Authors:** Lesly C. Ceniceros, John P. Capitanio, Erin L. Kinnally

**Affiliations:** ^1^California National Primate Research Center, University of California, Davis, Davis, CA, United States; ^2^Department of Psychology, University of California, Davis, Davis, CA, United States

**Keywords:** prenatal stress, infant behavior, resilience, temperament, rhesus monkey

## Abstract

The prenatal period is a developmental stage of peak sensitivity, during which environmental exposures can program post-natal developmental outcomes. Prenatal stress, in particular, has often been associated with detrimental neurobehavioral outcomes like mood and anxiety disorders. In the present study, we examined the effects of a stressful prenatal maternal experience (maternal relocation during pregnancy) on the post-partum development of offspring in rhesus macaques. To help isolate the effects of prenatal stress from genetic predispositions and post-natal experience, we compared biologically reared infants (infants raised with their biological mothers) with cross-fostered infants (those raised by non-related females in new social groups). We examined the effects of prenatal relocation stress on measures collected at 3–4 months of age during a standardized biobehavioral assessment. Unexpectedly, we found that prenatal stress resulted in a behavioral pattern consistent with resilience rather than anxiety: prenatal stress was linked with greater activity, lower anxiety, and more interaction with novel objects, as well as higher ratings of temperamental confidence during assessment. These effects were observed in infants reared by biological mothers as well as cross-fostered infants, suggesting that the effects of prenatal stress were not attributable to maternal genetics or post-natal factors. Our surprising results suggest that prenatal relocation stress may confer resilience in infant rhesus monkeys.

## Introduction

### Prenatal Stress and Functional Dysregulation

The prenatal period is a peak sensitivity period during which teratogens like drugs, alcohol, and stress, can shift infant development toward disadvantageous outcomes (Laegreid et al., 1989; Schneider et al., 2002; Ruiz and Avant, 2005). Maternal stress during pregnancy has been found to cause pro-inflammatory and stress axis changes that may permeate the placenta and reach the developing fetus (Ruiz and Avant, 2005). Chronic stress has also been observed to cause an over stimulation of the maternal hypothalamic-pituitary adrenal (HPA) axis, which results in excessive amounts of glucocorticoid production that can also cross the placenta barrier and potentially lead to emotional, intellectual, and neurodevelopment deficits (Gunnar, 1998). Some have reported that intense maternal prenatal stress, such as disruptive social changes, unfavorable living conditions, or exposure to substance abuse confers risk for behavioral and temperamental deficiencies like increased emotional reactivity, reduced exploration of the environment, and anxiety (Schneider et al., 2002; Richardson et al., 2006; Lester et al., 2009; Herrington et al., 2016). Others have demonstrated that prenatal stress can foster risk for externalizing behavior as children mature. For instance, Gutteling et al. (2005) found that mothers who reported a greater amount of perceived stress during pregnancy had toddlers who showed more problematic externalizing behavior, had greater amounts of disruptive temperament, and poorer attention regulation.

In contrast, some prenatal stressors seem to “inoculate” the developing infants against the harmful effects of stress by promoting resilience (DiPietro et al., 2006; Hartman et al., 2018; Serpeloni et al., 2019). DiPietro et al. (2006) showed that mild to moderate amounts of perceived stress and anxiety by the mother during pregnancy were associated with more advanced mental and motor development in human children at 2 years of age. A more recent report showed that prenatal stress may confer a protective phenotype, buffering animals against future stressors (Scott et al., 2017). Pregnant female mice were exposed to a series of chronic variable stressors during their last week of pregnancy. As adults, the prenatally stressed male offspring were housed in a naturalistic environment, with control male, and female counterparts. Prenatally stressed males were more often observed to occupy a subordinate social status as opposed to a dominant status, but the detrimental effects (significant weight loss, behavior inhibition: fleeing, hiding, incurring more wounds) associated with a subordinate status in control males were not observed. These data suggest that prenatal stress may promote resilience to social subordination stress in mice.

Some of the variability in the effects of prenatal stress in human studies may be attributed to complex interactions between prenatal stress and post-natal stress: prenatal socioeconomic status (Lobel et al., 1992), support systems (Dunkel-Schetter et al., 1996), or maternal attributes (DiPietro et al., 2006) each contribute to both the prenatal and post-natal environment. On the other hand, the experience of a prenatal stressor may change the post-natal environment a mother may provide. Animal models allow us to isolate genetic, prenatal, and post-natal influences on individuals through *cross fostering* infants to unrelated mothers in new social groups (Champoux et al., 1995; Francis et al., 1999; Kinnally et al., 2018). Non-human primates exhibit comparable reproduction and gestational characteristics to that of humans but in a reduced time frame making them excellent candidates for longitudinal studies (Schneider et al., 2002). Rhesus monkeys (*Macaca mulatta*) make an exceptional translational animal model of development due to their phylogenetic similarity to humans (Phillips et al., 2014).

### Current Study

The purpose of our study is to examine the effects of stressful prenatal events experienced by pregnant macaque females on the post-natal development of their offspring. Previous studies in macaques have examined the effects of other prenatal stressors (matrilineal overthrow: Herrington et al., 2016; chronic relocation with acoustic startle: Coe et al., 2003). Our opportunistic model of stress utilizes hospital relocations due to: injury, illness, or offspring related reasons for which the animal is temporarily removed from their home environment for an average of 9 days over an average of 1.5 relocations during pregnancy. This temporary relocation is a stressful, but common, event for outdoor housed captive rhesus macaques. Relocation requires temporary separation from family and peers and introduction into a novel environment, a procedure that animals will experience many times throughout their life course (Capitanio and Lerche, 1998; Dettmer et al., 2012; Hennessy et al., 2017). Permanent relocation to new social groups has been shown to increase HPA output and self-injurious behavior in the short- and long-term [1 year later: Davenport et al. (2008)]. Monkeys infected with SIV virus that are relocated frequently on a short-term basis exhibited decreased survival, perhaps resulting from this stress (Capitanio and Lerche, 1998). Most similarly to our short- term relocation model, Hennessy et al. (2014), observed a temporary, reversible depressive-like response in rhesus macaque adult males when relocated from outdoor cage housing to indoor individual housing for an 8-day period (Hennessy et al., 2014, 2017). Our study examines the association amongst exposure to intermittent short- term relocation stress during females' pregnancy and post-natal offspring behavioral development. We further investigated the role of genetic and post-natal factors in the effects of prenatal stress by comparing development in infants that remained with biological mothers with infants that were cross fostered to unrelated mothers in new social groups.

## Methods

### Experimental Subjects

One hundred eighty-eight infant rhesus macaques (89 males and 99 females) were raised with their biological or foster mothers in one of seven social groups housed in large outdoor enclosures that constitute the breeding colony of the California National Primate Research Center (CNPRC). Each field cage contained a large social group (80–150 members). These groups had similar social demographics, including 6–13 distinct matrilines with extended kin networks and animals of all age/sex classes. A subset of our animals (*N* = 57) were raised in specific pathogen free (SPF) enclosures. These groups were free of zoonotic diseases such as Herpes B. These groups had been originally founded with mostly nursery reared monkeys [for review, see Kinnally et al. (2018)]. However, all of our groups were intact for at least 3 years at the time of observation and on average, established 15 years prior to observation, so most of our subjects had themselves been born and raised in SPF enclosures, similar to non-SPF females. Eight of our SPF mothers had been nursery reared and 49 had been born and raised in our outdoor enclosures.

All social groups included 5–10 reproductively mature males, 25–60 reproductively mature females, and 25–75 subadult, juvenile or infant monkeys. Enclosures were 0.2 hectares with chain link fencing to allow visual access. Animals were fed monkey chow (LabDiet 5405) twice per day, once in the morning and once in the afternoon. All groups received fresh produce on a weekly basis. All procedures were approved by the UC Davis Institutional Animal Care and Use Committee and were in compliance with the National Institutes of Health Guide for the Care and Use of Laboratory Animals.

### Prenatal Relocation

We retrospectively extracted relocation data from CNPRC health records. Conception day was defined as 166 days (the macaque gestation period, Silk et al., 1993) before infant birth. All relocations of a pregnant female from the date of conception through date of infant birth were extracted. Relocations were defined as transitions from outdoor natal enclosures to indoor individual housing for a total of at least 24 h. There were three possible reasons females were relocated during pregnancy: injury, illness, or hospitalization of their current infant. Relocation trimester was calculated as: first trimester 0–55 days, the second trimester 55–110 days, and the third trimester 110–166 days. During relocation, the most common pharmacological treatments were ketamine (a sedative) and ketoprofen (a non-steroidal anti-inflammatory). Relocation lengths consisted of 1–36 days across 1–5 relocation bouts. Mean length of relocation days across all of the relocations was 9.25 days and the average number of relocations was 1.48 relocations. We used a dichotomous category for relocation because statistical analyses revealed that neither length, nor number of relocations, nor trimester of relocation, moderated the main effect of any relocation during pregnancy. Our prenatal stress exposure was therefore defined experiencing any relocation during pregnancy (*N* = 39), compared with females that were not relocated at any time during pregnancy (*N* = 149). See Table 1 for demographic comparisons between prenatal stress and control groups.

**Table 1:** Prenatal stress vs. control female demographics.

	**PS**	**Control**	***p***
	**(*n =* 39)**	**(*n =* 149)**	
Age	6.613	6.531	0.718
Prior pregnancies	2.260	2.210	0.533
Rank (rank/#females in group)	0.484	0.537	0.532
Maternal affiliation	0.303	−0.079	0.080
Maternal aggression	0.006	0.008	0.823
Maternal protectiveness	−0.257	0.104	0.160
Maternal neutrality	0.101	−0.053	0.245
Maternal sensitivity	0.088	−0.022	0.411
Number of days relocated	9.25	0.00	0.000
Reason for relocation (PS only)			
Illness—mother	4	NA	NA
Illness—infant	1	NA	NA
Injury—mother	34	NA	NA
Ketamine exposure	3.415	1.012	> 0.0001
Ketoprofen exposure	0.293	0.006	> 0.0001
Infant birth weight	0.986	0.936	0.100

### Cross-Fostering

Cross-fosters were selected to enhance genetic diversity between social groups. Post-partum mothers were identified on the morning of birth, and selected if they: (1) were unrelated to potential cross-foster mothers in other social groups, (2) had previously successfully reared a foster infant (all but one female adhered to this criterion) (3) had previously given birth to at least one infant. On post-natal Day 1, mother-infant dyads either remained undisturbed in outdoor enclosures (*n* = 146) or were relocated indoors for standardized cross fostering procedures (*n* = 42) that we have described in detail elsewhere (Kinnally et al., 2018, 2019). Briefly, dyads were removed from outdoor enclosures and transported to indoor procedure rooms. Mothers were sedated (10 mg/kg ketamine) and infants were removed by trained CNPRC staff and placed on the ventrum of the new foster mother who was also sedated. Dyads were monitored overnight to ensure that the foster mother accepted the infant after manipulation. Success of our cross-fostering procedure was 100%, with all foster mothers accepting new infants and returning to social groups within 24 h. Of our prenatal stress group, nine infants were foster reared and thirty were biologically reared. In our control group, thirty- three infants were foster reared and one hundred sixteen were biologically reared. Of our foster mothers, six had been prenatally relocated. Five of these mothers fostered offspring that had not been prenatally relocated and one fostered an infant which had been prenatally relocated.

### Mother-Infant Observations

Mother-infant dyads were observed in their social groups for 5 min per observation using focal dyadic sampling (Altmann, 1974), conducted one to four times weekly (range 1.25–4.25, average 2.27 observations per week), as we have described previously (Kinnally, 2014; Kinnally et al., 2018, 2019). Observations were conducted between 7:00 A.M. and 1:00 P.M., during post-natal weeks 1–12. Observation order was rotated daily so dyads were observed at different times of day across the prescribed observation period.

Mother-infant interactions were coded using a transactional coding system, describing the overall theme of an interaction from the perspectives of the initiator and the recipient (Lyons-Ruth et al., 1986; Kinnally et al., 2010; Kinnally, 2014). A transaction was defined as a change from one state of association that lasted 3 s or more, to a new state that was maintained for at least 3 s. Themes are defined in Table 2, and include protection, affiliation, neutral, rejection and aggression. Each dyadic interaction was characterized from the perspective of the mother and of the infant, and a theme assigned for each based on their behavior. For example, some common transaction types include: (1) infant approaches mother and initiates contact, mother initiates physical contact (which would be scored as Affiliative-Affiliative, received by the mother), (2) Mother retrieves infant and infant grabs mother's ventrum or back (Protective-affiliative), (3) Infant jumps on mother, mother swats the infant to the ground (Affiliative-aggressive, received by the mother).

**Table 2:** Maternal behavior transaction theme definitions.

Protective	Includes all behaviors intended to protect and restrict the infant's range of movement. The defining characteristic is the action of the mother pulling the infant toward her. Protectiveness is the only transaction that infants cannot engage in.
Affiliative	Includes behaviors including prosocial physical contact, such as grooming, licking, holding, and any other non-aggressive positive touch.
Neutral	Includes non-committal behaviors. The most common neutral transaction initiation is an approach without making physical contact. Neutral responses are those where the receiver does not react positively or negatively to an overture.
Rejecting	Includes behaviors that discourage interaction. The most common rejection theme includes walking away when mother approaches. This theme can only be a response to an overture.
Aggressive	Includes physical contact aggression. Includes scratching, hitting, biting, flattening, dragging, throwing, and any other physical contact that may inflict pain on the infant.

Adult raters were trained by a primatologist with experience in mother-infant interactions for at least eight 2-h training sessions. Rater reliability was determined at the end of the training period by calculating whether the rater was correct in recording each aspect of the transaction (maternal theme, infant theme, and recipient) for ten sequential 5-min trials. These trials were required to comprise of a wide variety of transaction types or were otherwise excluded from reliability calculations. Inter-rater reliability was 90% or better.

Rates of all transaction themes by the mother (Protective, Affiliative, Neutral, Rejecting or Aggressive) were calculated per observation period for analysis as we have described previously (Kinnally et al., 2010, 2018, 2019; Kinnally, 2014).

### Biobehavioral Assessment

At 90–126 days of age (mean 99.88 days), subjects were observed during a standardized biobehavioral assessment (BBA) at the CNPRC. During a 25-h relocation and separation from mothers and social groups, multiple behavioral (activity, emotionality, novel object interaction, temperament ratings), and physiological (plasma cortisol at four time points, selected to minimize impact on behavioral measures) measures were collected from infant subjects. The purpose of the biobehavioral assessment is to gauge infant individual differences under challenge conditions, when their mothers are not present to shape their behavior. The goal is to tap into the infant's biobehavioral organization—the structure of an individual's physiological and behavioral phenotype—that is intrinsic to them. Standardized procedures were designed to ensure that each subject had experiences comparable to all other subjects who underwent assessment. These procedures have been described in detail elsewhere (Golub et al., 2009; Kinnally et al., 2010; Capitanio, 2017). Standardized procedures included exposure to a single observer, both between tests and between years. The technician was unfamiliar with the animals before their arrival in our testing area for the BBA.

### Holding Cage Observations

Subjects were relocated from their outdoor enclosures to indoor individual housing (0.81 × 0.61 × 0.66 m) in a temperature-controlled room under a 12:12 h light/dark cycle by 9:00 a.m. At ~9:15 a.m., animals' activity- and emotion-related behaviors were recorded in a 5-min trial (see Table 3 for Ethogram). Two scales were identified using exploratory and confirmatory factor analyses on various sample subsets for these data from Day 1, as described in Golub et al. (2009). Factor analysis is a useful technique for identifying latent dimensions underlying multiple behaviors within or across contexts. Exploratory factor analysis identifies the structure of relationships between behaviors by assigning factor loadings. Factors that load higher than 0.4 are considered to contribute to the final factor score, which uses regression methods with factor loadings as beta weights to generate one factor score, which is a standardized score, or Z-score. Confirmatory factor analysis applies this structure to determine goodness of fit in replication datasets. Holding cage scales were labeled Activity because they loaded higher frequencies or durations of locomotion, eating, drinking, environmental exploration, crouching, and less hanging. Emotionality was so-named due to the high loadings of cooing, barking, scratching, threatening, and lipsmacking. Z-scores within year are used as outcome variables to control for year-to-year variation.

**Table 3:** Home cage and human intruder ethogram.

**States**
SI, sit: Hindquarters are on the floor; includes shifting weight slightly one step.
LI, lie: Relaxed posture with body resting on a horizontal surface.
ST, stand: Torso in a stationary position and weight is supported by 3 or 4 legs; can include steps taken that only involve one or two steps.
LO, locomote: Directed movement from one location to another.
CR, crouch: Ventral surface close to floor; head at or below shoulders.
SL, sleep: Eyes closed.
PA, pace: Repetitive rapid movement over the same path.
RO, rock/sway: Unbroken rhythmic movements.
HA, hang: Holding onto ceiling, sides or front of the cage; all 4 limbs off of floor.
**Events**
SC, scratch: Common usage.
CL, self-clasp: Hand or feet closed on fur or some body part.
SB, self-bite: Discrete biting action, usually directed to limbs.
SS, stroke: Gently bringing the hand or foot across the side of head or face.
SU, suck: Insertion into mouth of fingers, toes, and other body parts.
BF, back-flip: Tossing the body up and backwards in a circular motion in the air.
CJ, convulsive jerk: Sudden contractions of the limbs and trunk.
VC, coo: Medium-pitched, moderately intense, clear call.
VS, screech: Intense, very high-pitched.
VG, gecker: Staccato cackling sounds.
VB, bark: Gruff, abrupt, low-pitched vocalization.
LS, lip smack: Rapid lip movement usually with pursed lips, smacking sound.
TH, threat: Two or more of the following: open mouth stare, head bob, ear flaps, bark vocalizations.
FG, fear grimace: Exaggerated grin with teeth showing.
YA, yawn: Wide open mouth displaying teeth.
TG, tooth grind: Loud gnashing of teeth.
MS, motor stereotypy. Any of the following: Repeated movement, of a head flip, sway (side to side motion while standing or hanging), or up and down motion of the body.
EE, Environmental explore: Discrete manipulation by hand or mouth with the physical environment or objects in the cage.
ET, eat: Common usage.
DR, drink: Common usage.

### Human Intruder Trials

At ~2:00 p.m., each animal was tested in a single session comprising 4 1-min trials in a separate room from holding cages. The first trial (“Profile-Far”) involved the experimenter positioning herself 1 m in front of the animal's cage, and presenting her left profile to the animal for 1-min. At the end of the minute, the experimenter moved laterally toward the animal's cage, positioning herself to within 0.3 m from the front of the animal's cage, still holding the profile position (“Profile-Near”). One minute later, the experimenter returned to the 1 m location and made direct eye contact with the animal (“Stare-Far” condition). After 1 min, the experimenter moved to the near (0.3 m) position while maintaining eye contact. This position was also held for 1-min (“Stare-Near”). The same experimenter was used for all animals. Preliminary analysis showed that the best strategy for dealing with the four conditions that characterize this test (e.g., profile far, profile near, etc.) was to take a mean for each behavior across the four conditions. Exploratory and confirmatory factor analyses were done, and a four-factor solution was found (Gottlieb and Capitanio, 2013): Activity (active, cageshake, environment explore), Emotionality (convulsive jerk, fear grimace, self-clasp, vocal), Aggression (threat, vocal bark, vocal other), and Displacement (tooth grind, yawn). Z-scores within year are used at outcome variables.

### Novel Object Interaction

For the duration of the biobehavioral assessment, one of two small objects were present in the animal's housing at all times. Inside these objects was a recording device (Actiwatch, Philips Respionics, Andover, MA, USA) which is activated whenever a force is exerted on the object. First, a small (3.5″L × 1.5″D) black cylindrical shaped object weighing 0.090 kg, was placed in the infant's cage from the time the animal was relocated at 9:00 A.M. At ~4:15 P.M., a second white crayon shaped novel object of similar size (3.6″L × 1.5″D) weighing 0.085 kg and with the same Actiwatch recorder, was placed in the holding cage and remained until the end of assessment at 9:15 A.M. the following morning. Data from the recorders were parsed into the number of 15-s intervals for each 5-min period during which any force was exerted on the objects. Period 1 includes the time block from ~9:00 A.M to 12:15 P.M. on Day 1. Period 2 was defined as approximately the time between 12:15 P.M. and 4:30 P.M. Period 3 is defined as the overnight period between ~4:30 P.M. and 8:00 A.M. the next morning. Period 4 includes the time between 8:00 A.M. and 10:15 A.M.

### Temperament Ratings

Temperament data were collected by a trained observer at the end of the 25-h testing. The rater recorded her perception of each subject with regard to 16 adjectives describing temperament (e.g., confident, nervous, tense, timid), on a Likert scale of 1–7. Temperament assessments were performed by the same technician who had tested the animals in all of the other assessments. The technician specifically does not rate the animals until some time has elapsed between her last behavioral data collection and the ratings, in order to avoid having a recency effect on her memory of the animals. Because the temperament assessments are designed to reflect the technician's total experience with the animals during the 25-h period (i.e., not just while testing the animals individually, but also while handling during blood collection, performing husbandry procedures, etc.), getting inter-observer reliability on a regular basis is difficult, necessitating a second person accompanying the main technician across the entire testing period. When we have done this, we have found acceptable levels of inter- rater agreement and reliability. Finally, we note the values for the temperament measures are z-scored within each observation year, which can serve to minimize bias associated with technician “drift” over the years. We do note that the technician is highly experienced, having assessed all animals (*n* > 5,000) since the beginning of the BBA program in 2001, and has been involved with other studies that used a similar rating methodology. Exploratory and confirmatory factor analyses were applied to scores (Golub et al., 2009) on each of these adjectives to detect underlying dimensions of temperament. Four dimensions were found, which include Nervousness (including high scores on nervous, fearful, timid, and low scores on calm, confident), Confident (including high scores on confident, bold, active, curious, playful), Gentle (including high scores on gentle, calm, flexible, curious), and Vigilant (including high scores on vigilant, and low scores on depressed, tense, timid). Z-scores within year are used at outcome variables.

#### Plasma Cortisol

Procedures for blood sampling and drug treatment have been described in detail previously (Capitanio et al., 2005). Blood was sampled through femoral venipuncture four times over a 24-h period, and each sample was decanted into ethylenediaminetetraacetic acid-(EDTA) treated collection vials. The first sample was collected at 11:00 A.M. (AM sample), ~2.5 h following social separation/relocation. The second blood sample was collected ~5.0 h after the first sample at 4:00 P.M. (PM sample). Subjects were then immediately injected intramuscularly with dexamethasone (500 mg/kg; American Regent Laboratories, Shirley, NY, USA). The next blood sample was taken through femoral venipuncture 16.5 h later, at 8:30 A.M. (DEX sample). Immediately following blood sampling animals were injected with 2.5 IU ACTH (i.m. Organon, Inc., West Orange, NJ) and the final blood sample was collected 30 min later. Plasma cortisol was assayed in duplicate using commercially available kits (Diagnostics Products Corporation, Los Angeles, CA, USA). Interassay and intraassay coefficients of variance (calculated as the SD of relevant samples/mean of relevant samples) were < 10%.

### Data Analysis

Correlations among behavioral and physiological measures were conducted using Pearson's correlations. We conducted one multivariate analysis of variance (MANOVA) with sixteen dependent behavioral variables: four temperament factor scores (Vigilance, Confidence, Gentleness, Nervousness), holding cage Activity and Emotionality scores on Days 1 and 2, Novel object interaction during 4 binned time periods, human intruder factor scores (Activity, Emotionality, Aggression, and Displacement). We conducted an additional MANOVA with four dependent physiological variables: Predictors included prenatal relocation (present or absent) and foster status (cross-fostered or biologically reared). Covariates included maternal age, the mother's number of previous pregnancies, maternal social hierarchy rank (absolute rank divided by the total number of adult females in the social group), infant sex, and four maternal care factors: maternal protectiveness, affiliation, neutrality, and aggression. Roy's Largest Root was used for reporting multivariate tests. *Post-hoc* testing for individual dependent variables was conducted using individual F-tests.

## Results

**Figure 1:**
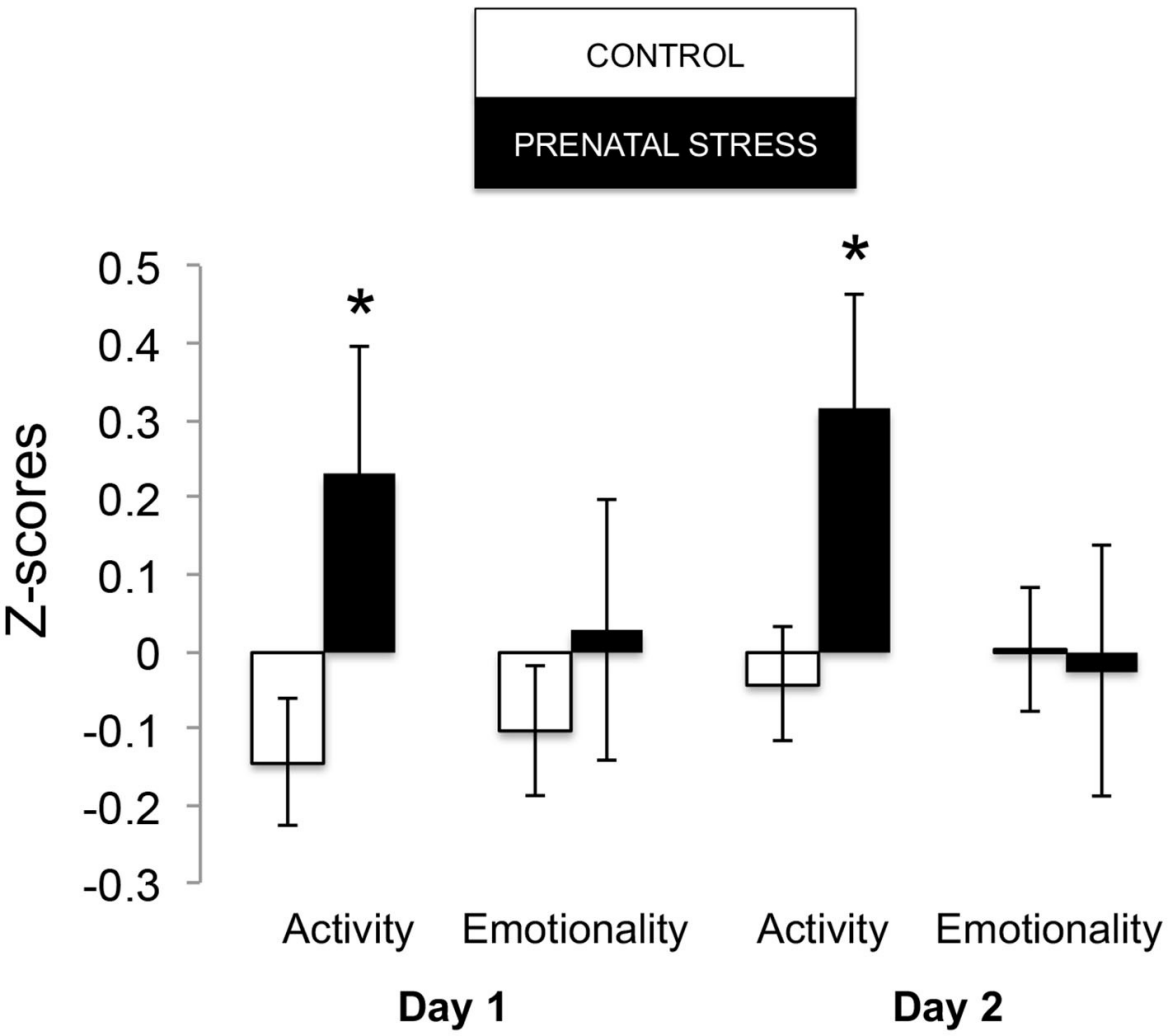
Effects of prenatal stress on Holding cage Activity and Emotionality during biobehavioral assessment between offspring of prenatally stressed mothers (*n* = 39) and control mothers (*n* = 149). Means are presented ± standard error of the mean. *p < 0.05.

**Figure 2:**
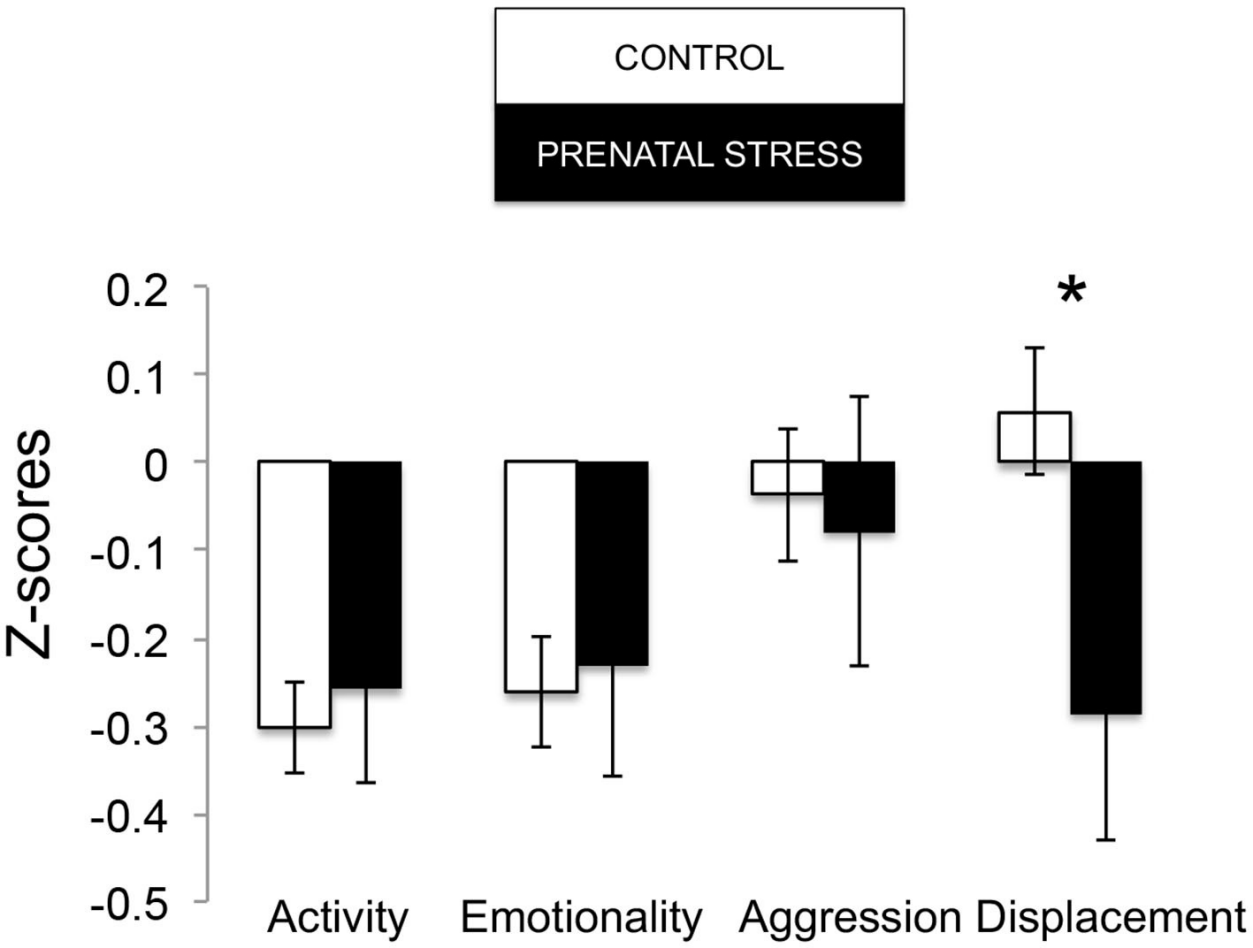
Human Intruder response during biobehavioral assessment between offspring of prenatally stressed mothers (*n* = 39) and control mothers (*n* = 149). Means are presented ± standard error of the mean. *p < 0.05.

**Figure 3:**
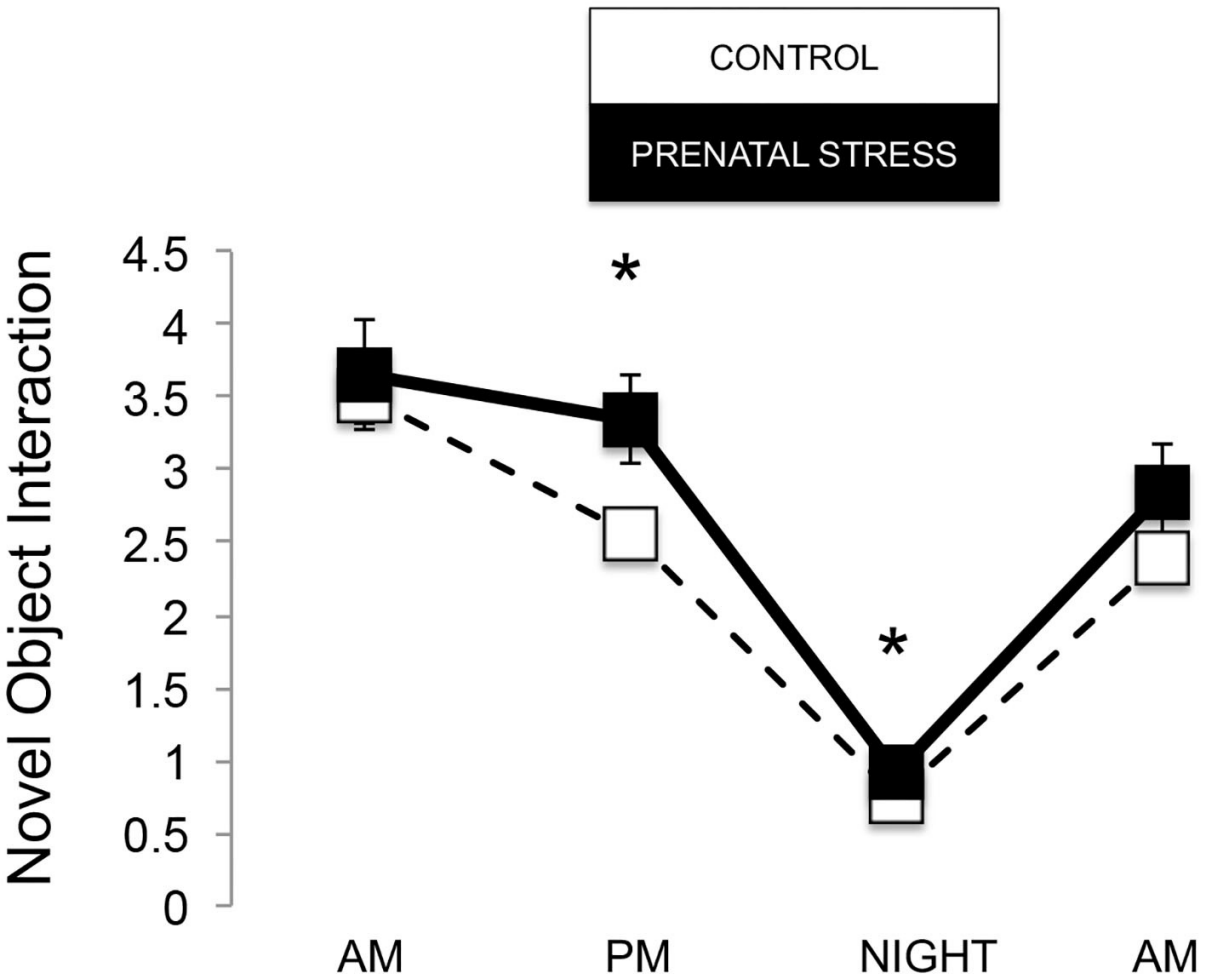
Novel object interaction during biobehavioral assessment between offspring of prenatally stressed mothers (*n* = 39) and control mothers (*n* = 149). Means are presented ± standard error of the mean. *p < 0.05.

**Figure 4:**
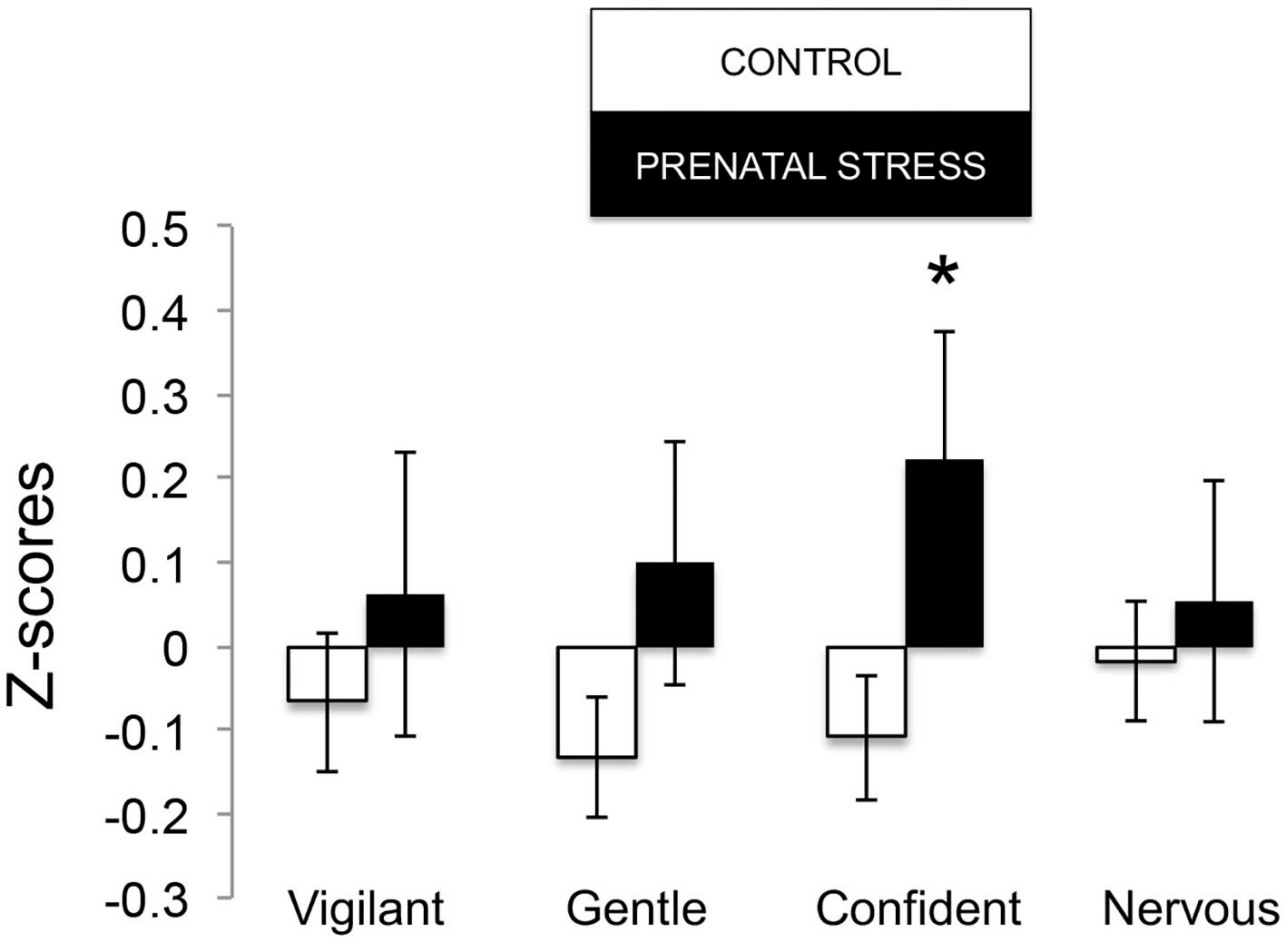
Temperament measures during biobehavioral assessment between offspring of prenatally stressed mothers (*n* = 39) and control mothers (*n* = 149). Means are presented ± standard error of the mean. *p < 0.05.

**Figure 5:**
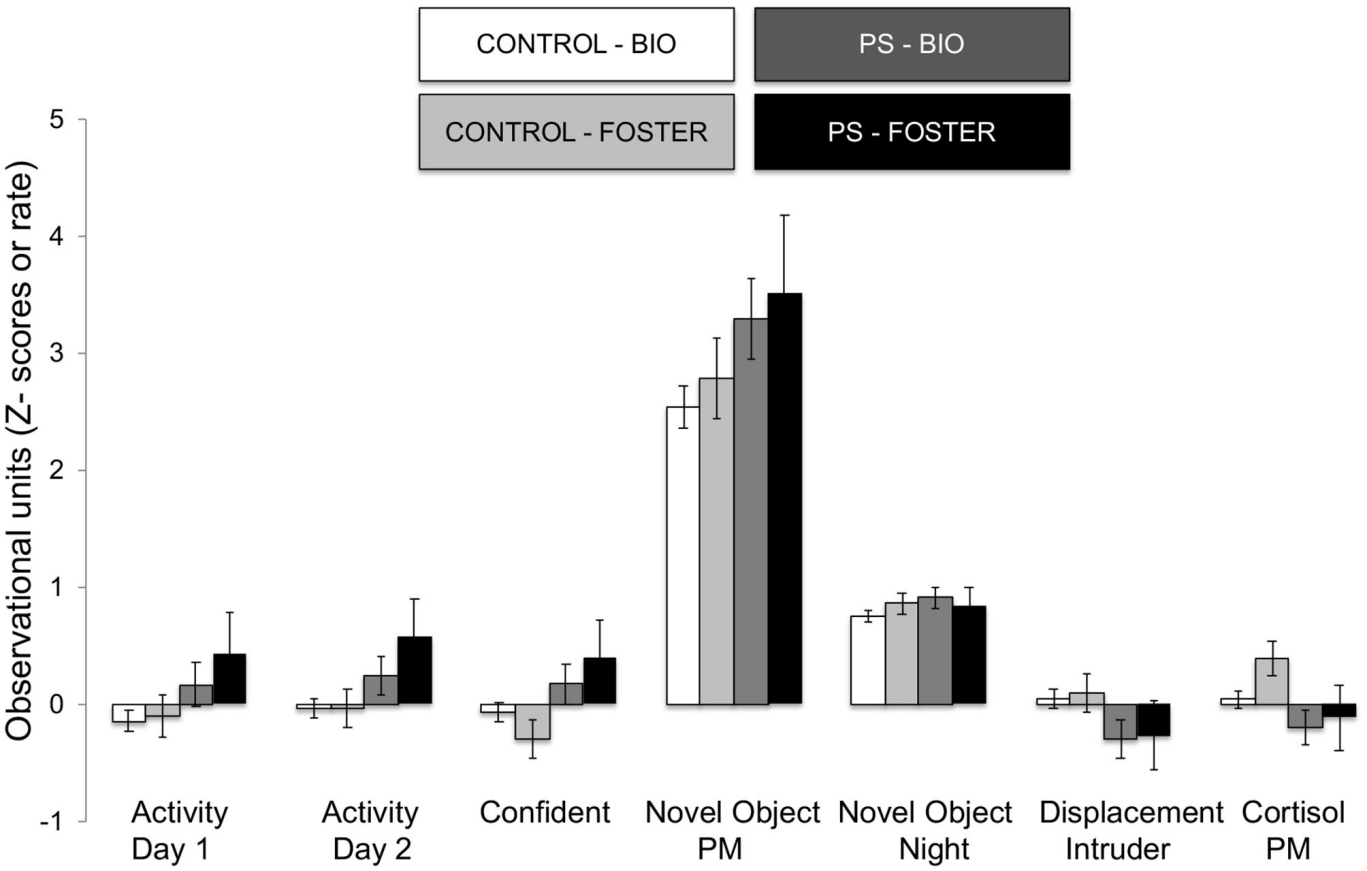
Effects of prenatal stress on significant temperament measures during biobehavioral assessment in biologically reared and cross-fostered infants. Means are presented ± standard error of the mean. CONTROL = No prenatal stress (*n* = 149) PS = Prenatal Stress (*n* = 39), BIO = infants raised by biological mothers (*n* = 146), FOSTER = infants raised by unrelated foster mothers (*n* = 42). *p* < 0.05.

The omnibus MANOVA model showed that maternal relocation during pregnancy significantly predicted biobehavioral organization across each of our assessments [Roy's Largest Root = 0.173, power = 0.888, F_(16, 174)_ = 1.722, *p* = 0.047, partial eta^2^ = 0.148]. Prenatally stressed infants exhibited higher Activity scores on Day 1 of assessment [*F*_(16, 174)_ = 3.898, *p* = 0.05, partial eta^2^ = 0.022] as well as Day 2 of assessment [*F*_(16, 174)_ = 4.380, *p* = 0.036, partial eta^2^ = 0.025], in comparison to counterparts whose mothers had not been relocated during pregnancy (see Figure 1). Prenatal relocation was also associated with lower Displacement scores in the human intruder test [*F*_(16, 174)_ = 4.414, *p* = 0.037, partial eta^2^ = 0.025; see Figure 2]. Prenatally relocated offspring also exhibited significantly higher rates of interaction with a novel object during period 2 [*F*_(16, 174)_ = 5.35, *p* = 0.022, partial eta^2^ = 0.03], and period 3 of assessment [*F*_(16, 174)_ = 4.086, *p* = 0.045, partial eta^2^ = 0.023; see Figure 3]. Prenatal relocation was also linked with higher Confidence scores at the trend level [*F*_(16, 174)_ = 3.713, *p* = 0.056, partial eta^2^ = 0.021; see Figure 4]. Foster status was not related to any measure [*F*_(16, 174)_ = 0.541, *p* = 0.922, partial eta^2^ = 0.05], as a main effect or in interactions with prenatal stress [*F*_(14, 173)_ = 0.866, *p* = 0.609, partial eta^2^ = 0.081; see Figure 5].

No significant differences in biological vs. foster mothers were observed in any maternal behavior measure (MANOVA F = 1.089, *p* = 0.363).

Of all maternal and infant factors considered in our model, only maternal rank [Roy's Largest Root = 0.164, F_(16, 174)_ = 1.948, *p* = 0.020, partial eta^2^ = 0.164] and SPF status [Roy's Largest Root = 0.187, F_(16, 174)_ = 1.858, *p* = 0.028, partial eta^2^ = 0.158] predicted overall biobehavioral organization. The effects were such that offspring of lower ranked females exhibited significantly greater Activity on Day 1 [*F*_(1, 174)_ = 5.379, *p* = 0.022, partial eta^2^ = 0.030], greater Emotionality on Day 1 [*F*_(16, 174)_ = 7.455, *p* = 0.007, partial eta^2^ = 0.041], and greater Emotionality on Day 2 [*F*_(16, 174)_ = 4.29, *p* = 0.04, partial eta^2^ = 0.024]. Lower ranked females had offspring that were rated as more vigilant [*F*_(16, 174)_ = 4.216, *p* = 0.042, partial eta^2^ = 0.024]. SPF infants were significantly less active on Day 1 [*F*_(16, 174)_ = 7.729, *p* = 0.006, partial eta^2^ = 0.043], and interacted with a novel object less often during Period 2 [*F*_(16, 174)_ = 10.234, *p* = 0.002, partial eta^2^ = 0.056], Period 3 [*F*_(16, 174)_ = 6.391, *p* = 0.012, partial eta^2^ = 0.035], and Period 4 [*F*_(16, 174)_ = 4.396, *p* = 0.037, partial eta^2^ = 0.025]. SPF infants exhibited higher Emotionality [*F*_(16, 174)_ = 4.445, *p* = 0.036, partial eta^2^ = 0.025] and Aggression scores [*F*_(16, 174)_ = 4.04, *p* = 0.046, partial eta^2^ = 0.023] in response to a human intruder. *Post-hoc* testing revealed that the only maternal behavior factor that predicted infant behavior was Neutral mothering. Infants that received higher rates of neutral overtures and response from mothers were more active on Day 1 [F_(16, 174)_ = 6.47, *p* = 0.011, partial eta^2^ = 0.036] and more gentle [*F*_(16, 174)_ = 7.407, *p* = 0.007, partial eta^2^ = 0.041]. Infants of prenatally relocated mothers did not differ in birth weight (*t* = 1.635, *p* = 0.100), and means of prenatally relocated infants were on average higher (0.986 vs. 936 kg, respectively).

**Figure 6:**
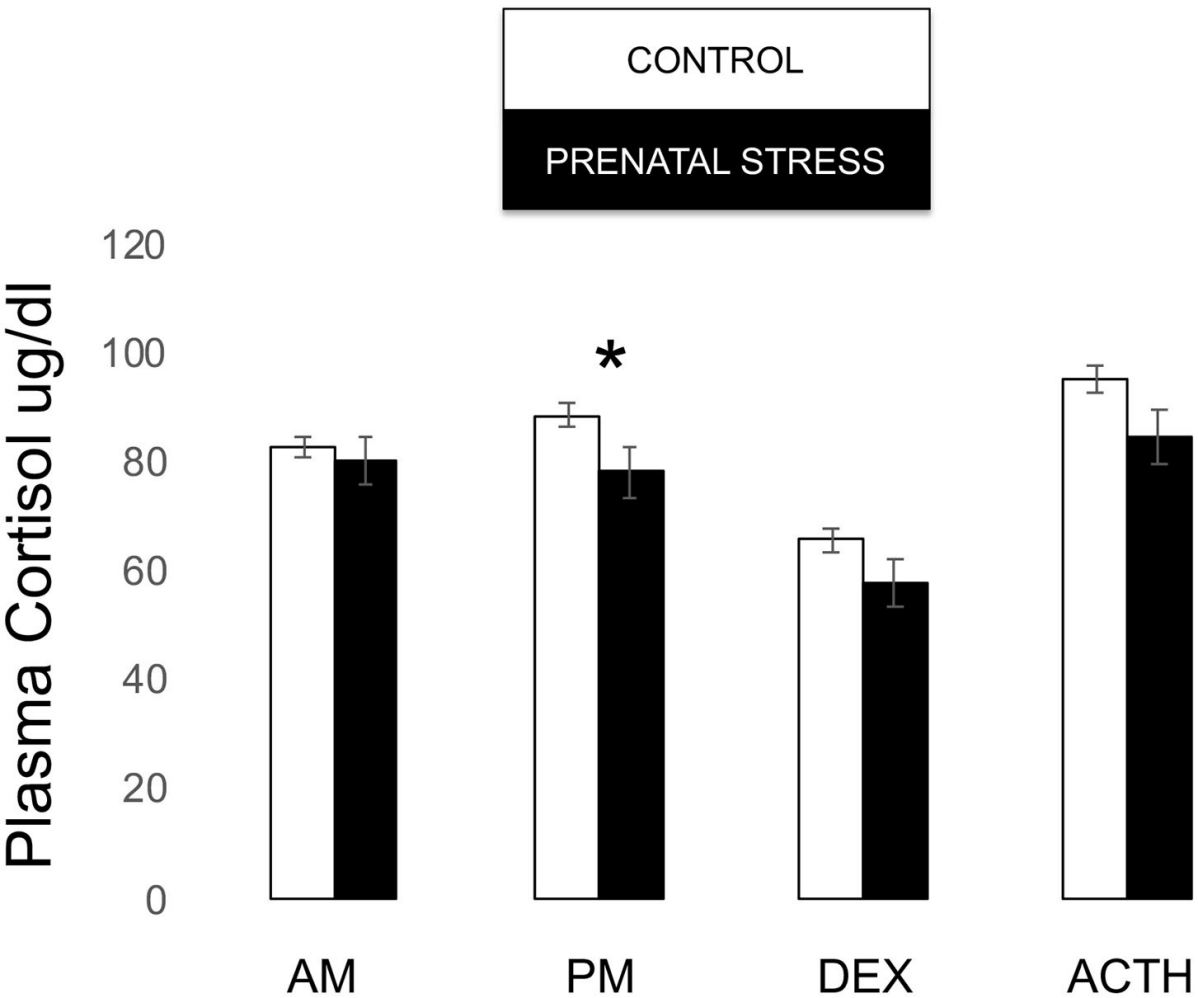
Plasma cortisol measures during biobehavioral assessment between offspring of prenatally stressed mothers (*n* = 39) and control mothers (*n* = 149). Means are presented ± standard error of the mean. *p < 0.05.

Though our omnibus model did not reveal an overall effect of relocation on infant cortisol [*F*_(1, 171)_ = 1.381, *p* = 0.243], *post-hoc* tests revealed that prenatal stress was associated with lower cortisol both in the post-stress samples [*F*_(1, 171)_ = 4.094, *p* = 0.045] and at the trend level post-ACTH [*F*_(1, 171)_ = 3.429, *p* = 0.066; see Figure 6].

Biobehavioral measures across conditions were moderately correlated, as we have previously reported (Kinnally et al., 2010; Supplementary Table 1).

## Discussion

The consequences of prenatal stress on infant development are diverse (Clarke and Schneider, 1993; Gutteling et al., 2005; DiPietro et al., 2006; Hartman et al., 2018). Some studies have shown prenatal stress leads to internalizing outcomes (negative affect, poor soothability, anxiety; Gutteling et al., 2005; Hentges et al., 2019), some to externalizing outcomes (impulsivity, poor self control; Gutteling et al., 2005; Betts et al., 2014; MacKinnon et al., 2018), and still others, to resilient outcomes (advanced cognitive development and stress resistance; Fujioka et al., 2001; DiPietro et al., 2006). We observed that offspring of mothers that were relocated for an average of 9 days over an average of 1.5 relocations during pregnancy were more active, confident, and willing to engage with novelty, and exhibited lower post-stress cortisol during a moderately stressful social separation at 90–120 days of age. Controlling for the genetic and post-natal confounds of prenatal stress introduced when mothers rear their own biological offspring, we compared biologically reared infants with infants cross-fostered to new mothers. Intriguingly although our cross- fostered sample size was small, the notable behavioral consequences were identical across both groups, demonstrating the independence of prenatal stress effects from genetic confounds or post-natal maternal factors.

Prior research suggests that prenatal stress is a risk factor for behavioral dysregulation and problematic outcomes in animal and human health (Schneider et al., 1992, 2002; Coe et al., 2003; Gutteling et al., 2005; Ruiz and Avant, 2005; Pryce et al., 2011). In studies conducted on human children, prenatal stress has been linked with both internalizing and externalizing behavior dysregulation (Betts et al., 2014; MacKinnon et al., 2018; Hentges et al., 2019). Moreover, multiple studies have shown a link between prenatal stress and signs of internalizing behavior at an early age. Internalizing behaviors include negative affect, anxiety, depression, and withdrawn behavior (Gutteling et al., 2005; Hentges et al., 2019). For example, in a study measuring the effects of prenatal stress on 6-month-old human infants, prenatal stress and anxiety were found to predict higher levels of negative emotional affect thus increasing the risk for later internalizing behavior (Nolvi et al., 2016). Similarly, Hentges et al. (2019) found that indices of prenatal stress such as perceived stress, anxiety, and depression were indicative of greater child internalizing behavior at ages 3 and 5 years of age. In addition, prenatal stress has also been found to amplify interaction effects amongst a challenging post-natal environment by predicting greater child internalizing behaviors when exposed to greater amounts of prenatal stress (Hartman et al., 2020). Another body of work shows that as children mature, externalizing behavior such as conduct problems and poor impulse control, may emerge (Van den Bergh and Marcoen, 2004; Rice et al., 2010; MacKinnon et al., 2018). However, in the majority of these studies, the link between prenatal stress and externalizing phenotypes is typically not apparent until late childhood or adolescence. Moreover, these studies did not control for post-natal confounds on offspring development. Behaviorally, our monkeys represent a useful model to track these links: in our monkeys, we observed similar patterns of temperamental tradeoffs: externalizing traits (activity, novel object interaction, confidence) were moderately intercorrelated and these traits were inversely associated with internalizing traits (nervousness).

Because the age of our infants (3–4 months) is equivalent to about 1 year of human age, we expected our results to align with younger human children, which largely show signs of internalizing behavior following prenatal stress (Nolvi et al., 2016; Hartman et al., 2020). But prenatal relocation stress did not predict internalizing behavior in our infants. On the contrary, prenatally relocated animals exhibited more active, exploratory, and confident behavior. Upon brief separation from mothers and social groups, within 3 h, prenatally relocated infants engaged in more active exploration of their novel indoor housing, displaying activities such as locomotion, eating, drinking, environmental exploration, and crouching. At the same time during assessment, they did not exhibit differences in emotion-related behavior, which includes cooing, barking, scratching, threatening, and lipsmacking. This pattern continued into the next day, when prenatally relocated infants showed more activity and exploration, but not greater emotionality, which suggests that these infants showed consistent curiosity about, rather than fear of, their temporary environments. Prenatally relocated infants also showed reduced anxiety (displacement behaviors tooth grinding and yawning) in response to a human intruder challenge (a human standing in front of their holding cage at near and far distance, and making eye contact or showing a side profile) compared with Control infants. Human intruder measures were strongly correlated across Profile and Stare conditions and so combined into one measure as we have described previously (Kinnally et al., 2010). Throughout the afternoon and through the night of assessment, prenatally relocated infants interacted more often with novel objects. Finally, at the end of the 25-h assessment, relocated animals were rated as more confident (curious, playful, confident, bold, active) than Control infants. This pattern of behavior suggests an underlying engagement with the environment, and reduced anxiety, during stress. These results, on one hand, may translate to human externalizing behaviors like impulsivity and behavioral disinhibition. Consistent with this idea, we have previously shown that adult macaques that interact with novel objects more quickly (similar to infants in the present study) are more impulsive and aggressive in social contexts, consistent with an externalizing phenotype (Kinnally et al., 2008).

However, we suggest that the pattern of behaviors displayed by prenatally relocated infants may suggest a pattern more consistent with a robust, engaged, and curious response to challenge, rather than an impulsive response. In the face of a novel stressful challenge, these animals adapted well and interacted with their environment positively and flexibly. There is support for the idea that behavioral patterns like these are associated with positive engagement and adaptive stress response in rhesus monkeys. Infant macaques' subjective well-being (rated by trained observers) was positively correlated with greater characteristics of openness, and assertiveness, and negatively correlated with signs of anxiety (Simpson et al., 2019). We have previously shown that while rapid interaction with novelty may suggest impulsivity and adverse social outcomes, sustained and exploratory interest in novelty favors positive social behavior and relationships (Kinnally et al., 2008). In humans, such positive emotionality is associated with greater physical and mental health, such as faster cardiovascular recovery from exposure to stressful conditions (Fredrickson and Levenson, 1998; Lyubomirsky et al., 2005). Interestingly, these personality traits used to distinguish trait- resilient individuals appear to establish in early infancy and maintain their positive correlation with subjective well-being well into adulthood providing signs of early adaptive coping (Tugade et al., 2004). Our results suggest that animals that were relocated while *in utero* may actually be more resilient when later dealing with challenge. This interpretation would be consistent with a prenatal stress inoculation effect, consistent with some human and animal studies (DiPietro et al., 2006; Ehrlich and Rainnie, 2015; Scott et al., 2017; Serpeloni et al., 2019).

Further, our cortisol data suggest that prenatally relocated infants show a lesser cortisol stress response compared with offspring of mothers that were not relocated. Previous studies in macaque prenatal stress have shown the opposite: that prenatal stress increases cortisol output in offspring (Coe et al., 2003), which as we have described, correlated with more anxious behavior. Our combined results point to the opposite effect on our monkeys following prenatal relocation stress, enhancing behavioral and physiological resilience to challenge in infancy.

Our data are consistent with recent syntheses that suggest that prenatal stress can have context dependent influences on individual development (Pluess and Belsky, 2011; Cymerblit-Sabba et al., 2013; Hartman et al., 2018). We suggest that commonplace prenatal stress may confer stress inoculation, while more intense stressors may confer risk (Glover et al., 2010). For example, previous studies utilizing a combined effect of prenatal relocation and stress were arguably somewhat intense—i.e., relocating a female into a darkened room daily for 1 week every 5 weeks, and introducing unpredictable acoustic startle during relocation (Coe et al., 2003). Our temporary relocation may have been relatively mild, as adult females had experienced similar relocations for similar reasons multiple times in their lives. However, because all animals were relocated for reasons that may have added to their stress, like injuries inflicted by other group members, illness, or health problems of their infant, it is possible that relocation was more than a commonplace stressor for our subjects. Our results suggest that our stressor differs from previous experimental stressors in terms of their influence on infants. In Coe et al. (2003), prenatally stressed infants were less engaged with their environment than control infants, while our relocation stress was linked with more engagement with the environment, confidence and reduced anxiety. One reason that our stressor may be considered less intense is because of its ecological relevance: our stressor potentially equates to normative stress naturally encountered throughout macaque (and possibly human) pregnancy. Yet another possibility is that the reason for relocation played a larger role than we were able to detect: the majority of our mothers were relocated due to injuries while a subset of five females were relocated due to illness. Alternatively, the harmful effects caused by the relocation stressor may have also been ameliorated through social buffering when reuniting with friends and family in their home enclosures. Though our preliminary analysis of differences in offspring of females relocated due to illness vs. injury yielded no significance, caution should be taken in interpretation due to our smaller subsample. It is possible that relocation due to injury is a very different experience than relocation due to illness.

Some of the variability in the effects of prenatal stress in human studies may be attributed to correlations with post-natal factors: socioeconomic status (Lobel et al., 1992), a steady support system (Dunkel-Schetter et al., 1996), or differences in individual maternal attributes (DiPietro et al., 2006) that influence pre- and post-natal development in humans. Translational animal models of prenatal stress are useful to compare the relative effects of genetics, prenatal and post-natal experiences on the developing infant and disentangling nature vs. nurture effects. We compared the effects of our prenatal relocation stressor on infants reared by their biological mothers vs. foster mothers to control for social/environmental influences and ensure that our results were not due to genetic confounds between the mother and offspring (Kinnally et al., 2019). We observed that behavioral effects of prenatal stress were consistent between foster and biologically reared infants, suggesting that prenatal stress had a unique effect on post-natal development independent from interactions with maternal behavior or genetic confounds. This is consistent with a genetically informed human prenatal stress study that compared infants born via *in vitro* fertilization (Rice et al., 2018). Infants born of eggs donors that had experienced prenatal stress but gestational mothers that had not experienced stress showed higher risk for antisocial behavior and anxiety (Rice et al., 2018). Taken together, our results suggest that prenatal stress effects were specific and not mediated by genetic or post-natal correlations with prenatal stress that might arise due to maternal anxiety or chronic environmental stress in the social group.

Maternal factors are to be considered an important direct contributor to an infant's indirect prenatal and direct post-natal environment. Though post-natal factors did not appear to confound our prenatal stress effects, as we might expect, some maternal characteristics influenced infant biobehavioral development. Features of the social environment and maternal care played a role in infant biobehavioral development. In our study, lower maternal rank predicted higher activity and emotionality on Day 1 with continued emotionality into Day 2. Offspring of lower ranked mothers were also rated as more vigilant. This is consistent with the notion that the social environment of lower ranked mothers heightened awareness and emotionality in infant offspring early in life, wither due to the infant's direct experience in the social group or indirect effects through the mother's behavior or physiology. We also observed a global effect of SPF status on biobehavioral development. SPF enclosures are defined by at least two features: they were founded with so-called nursery reared (NR) founders to breed out zoonotic diseases, and they house monkeys free of these specific pathogens. NR entails separation from mothers at birth and rearing in a socially restricted environment. In adolescence, infants are moved to large SPF social groups. In the present study, our SPF infants showed more emotionality, less interaction with novel objects and more intense reactions to a human intruder. The results are consistent with a more anxious phenotype. We have previously shown that paternal line descendants of NR males show increased levels of anxiety (Kinnally et al., 2018), so the fact that SPF animals likely have more NR ancestors may explain this effect. While there were no global effects of maternal behavior in the omnibus model, *post-hoc* tests showed that neutral mothering predicted more activity on Day 1, higher ratings of temperamental gentleness and less temperamental nervousness. Neutrality in maternal care is used to describe dyadic interactions in which the infant initiates a proactive interaction toward the mother and the mother maintains proximity but does not respond in any particular proactive manner (Kinnally et al., 2018). Each of these maternal factors were controlled for in the overall analysis, and were independent of prenatal stress effects. These results highlight the importance of complex post-natal maternal factors on infant development, in addition to and independent of commonplace prenatal stress.

A major limitation of this study is that we cannot infer direct causality on the effects of prenatal stress because we did not specifically manipulate relocation stress. Since our prenatal relocation data were retrospective and opportunistic, we were not able to control for potential mediating factors related to aspects of relocation, such as reason for relocation or length of stay. For example, most females that were relocated were relocated due to injuries incurred in the social group, and relatively fewer due to illness. We did control for these variables in our early analyses and did not find a significant correlation between reason for relocation or length of relocation stay and our outcomes. However, it is possible that behaviors that increase risk for injury in social groups may be correlated with other life stressors. We also cannot entirely preclude that correlational factors associated with relocation may have contributed to our effects. For example, we examined whether the number of ketamine exposures, which was higher in prenatally relocated females, explained the effects of prenatal relocation, but the number of prenatal ketamine exposures alone did not drive our behavioral effects. But because all prenatally relocated females were treated with ketamine (as were most control females, during regularly scheduled health checks) during pregnancy, we cannot preclude an interaction effect between ketamine and relocation. Some studies have reported an effect of neuronal loss, cognitive deficits, and depressive-like offspring behavior as a result of maternal exposure to ketamine during prenatal stages (Zhao et al., 2014). Although further exploration is needed to investigate neural effects of ketamine in our subjects, our results did not align with the behavioral irregularities observed in these studies. Moreover, the likelihood that ketamine specifically confounds our prenatal stress condition seems remote, as additional studies have linked ketamine (and combined genetic factors) with biobehavioral outcomes suggesting poorer attention and internalizing behavior (Capitanio et al., 2012), while we believe our results point to resilient outcomes arising from prenatal manipulation. However, ketamine exposure has been linked with antidepressant effects in the exposed individual (Berman et al., 2000), so it remains possible that that prenatal ketamine played a role in the resilient outcomes we observed. This notion is somewhat undermined by our finding that neither the reason for relocation, nor number of ketamine exposures were statistically linked with our outcomes, this will be an important experimental control in future studies. Another possibility is that prenatal stress shifted aspects of development that we did not capture that impacted our results. For example, if prenatal stress led to premature birth, this could have affected our results. We do not believe this was the case, however, as our prenatal stress group did not differ in birth weight from controls. Our results however suggest that experimental manipulation of commonplace stress during pregnancy may yield a useful monkey model of enhancing stress resilience during infant neurodevelopment.

In conclusion, our findings support the notion that stress generated through maternal relocation during pregnancy led to a more active, confident and engaged phenotype in infant rhesus monkeys. One interpretation is that this commonplace prenatal stressor conferred resilience to prenatal stress. These findings highlight a need for future work (particularly experimental work) to address how mild prenatal stress may optimize behavioral stress response. Identifying these thresholds could help pave the way in exploring intervention options in cases of highly adverse behavioral outcomes.

## Data Availability Statement

The original contributions presented in the study are included in the article/Supplementary Material, further inquiries can be directed to the corresponding author/s.

## Ethics Statement

The animal study was reviewed and approved by UC Davis Institutional Animal Care and Use Committee.

## Author Contributions

LC: conceived of and executed study and wrote paper. JC: collected biobehavioral data, edited paper, and funded research. EK: senior author, statistical analyses, edited paper, and funded research. All authors contributed to the article and approved the submitted version.

## Conflict of Interest

The authors declare that the research was conducted in the absence of any commercial or financial relationships that could be construed as a potential conflict of interest.
